# The Microbes of Seborrhœa and of Acne

**Published:** 1897-05-01

**Authors:** 


### THE MICROBES OF SEBORRHŒA AND OF ACNE.

From many points of view considerable interest attaches to M. Sabouraud's recent researches into the bacteriological flora of the hairy scalp, not merely as showing the manner in which baldness in its various forms is produced (which has already been described in The Hospital), but as illustrating certain points in regard to the bacteriology of morbid states which appear sometimes to be overlooked. If Sabouraud's opinion is correct—and he is essentially careful to keep his theories well within the bounds marked out by his facts—seborrhœa, alopecia areata, and the gradual loss of hair leading to ordinary baldness are all infective diseases produced by the growth and chemical activity of one and the same bacillus. In alopecia this activity is extreme, it may be even fulminating; in baldness it is moderate, but the germ is endemic in enormous quantities. The experimental proof of this is not yet complete, nor does M. Sabouraud claim that his statement is of a final nature; still the results, as far as they have gone, are extremely important, and open up a yet wider field for future investigation. The bearing of this research upon some previously described bacteriological investigations is important. Sabouraud seems to have proved conclusively that the acne bacillus of Unna and Hodara is neither restricted in its occurrence to the acne pustule nor the cause of the disease when its presence is indubitable. This bacillus is apparently identical with his own microbe above referred to, and is therefore, as far as is at present known, really accidental in the comedones. Sabouraud's views on this point confirm the general opinion of dermatologists who have hesitated to accept Unna and Hodara's micro-organism as the cause of a disease so obviously related to infection with pyogenic cocci as is acne.

